# Cognitive and psychosocial development of HIV pediatric patients receiving highly active anti-retroviral therapy: a case-control study

**DOI:** 10.1186/1471-2431-10-99

**Published:** 2010-12-27

**Authors:** Loretta Thomaidis, Georgia Bertou, Elena Critselis, Vassiliki Spoulou, Dimitrios A Kafetzis, Maria Theodoridou

**Affiliations:** 1 Developmental Assessment Unit, Second Department of Pediatrics, "P. & A. Kyriakou" Children's Hospital, National and Kapodistrian University of Athens School of Medicine, Athens, Greece; 2 Second Department of Pediatrics, "P. & A. Kyriakou" Children's Hospital, National and Kapodistrian University of Athens School of Medicine, Athens, Greece; 3 First Department of Pediatrics, "Aghia Sophia" Children's Hospital, National and Kapodistrian University of Athens School of Medicine, Athens, Greece

## Abstract

**Background:**

The psychosocial development of pediatric HIV patients has not been extensively evaluated. The study objectives were to evaluate whether emotional and social functions are differentially associated with HIV-related complications.

**Methods:**

A matched case-control study design was conducted. The case group (n = 20) consisted of vertically infected children with HIV (aged 3-18 years) receiving HAART in Greece. Each case was matched with two randomly selected healthy controls from a school-based population. CNS imaging and clinical findings were used to identify patients with HIV-related neuroimaging abnormalities. The Wechsler Intelligence Scale III and Griffiths Mental Abilities Scales were applied to assess cognitive abilities. The age specific Strengths and Difficulties Questionnaire was used to evaluate emotional adjustment and social skills. The Fisher's exact test, student's t-test, and Wilcoxon rank sum test were used to compare categorical, continuous, and ordinal scores, respectively, of the above scales between groups.

**Results:**

HIV patients without neuroimaging abnormalities did not differ from patients with neuroimaging abnormalities with respect to either age at HAART initiation (p = 0.306) or months of HAART treatment (p = 0.964). While HIV patients without neuroimaging abnormalities had similar cognitive development with their healthy peers, patients with neuroimaging abnormalities had lower mean General (p = 0.027) and Practical (p = 0.042) Intelligence Quotient scores. HIV patients without neuroimaging abnormalities had an increased likelihood of both Abnormal Emotional Symptoms (p = 0.047) and Hyperactivity scores (p = 0.0009). In contrast, HIV patients with neuroimaging abnormalities had an increased likelihood of presenting with Abnormal Peer Problems (p = 0.033).

**Conclusions:**

HIV patients without neuroimaging abnormalities are more likely to experience maladjustment with respect to their emotional and activity spheres, while HIV patients with neuroimaging abnormalities are more likely to present with compromised social skills. Due to the limited sample size and age distribution of the study population, further studies should investigate the psychosocial development of pediatric HIV patients following the disclosure of their condition.

## Background

Untreated HIV infection among children has been associated with childhood cognitive, motor, language, and psychological developmental deficits [1,2]. Such developmental deficits are attributed to central nervous system disease involvement, including HIV-related neuroimaging abnormalities. Mental and motor developmental delays may commence at as early an age of 4 months, and may present either as selective or global neurodevelopmental delays [3]. Moreover, the consequent neuropsychological deficits have been associated with the severity of HIV-related neuroimaging abnormalities [4-7].

The introduction of novel antiretroviral therapies has led to marked improvements regarding the survival and consequent quality of life of HIV seropositive children [8-10]. Recent advances in the management and treatment of HIV through the administration of Highly Active Antiretroviral Therapy (HAART) have dramatically improved the disease prognosis among pediatric patients. HAART has been associated with sustained immune function and suppressed HIV viral load, as well as the consequent diminishment of secondary infections and malignancies, thus contributing to the prolonged survival of patients [9,11,12].

As a result of the effectiveness of HAART management, pediatric HIV patients have an enhanced survival, albeit with a chronic condition, that may present with related social, physical, and psychological challenges affecting their development [12,13]. Thus, HIV pediatric patients may have an elevated risk of presenting with psychosocial problems, including conduct problems and social maladjustment [3,7], similar to those observed among other chronically ill pediatric patients [14,15]. It is thought that both the frequency and severity of such problems may be most prominent among those children with HIV-related complications, as observed among patients with neuroimaging abnormalities [16,17].

The purposes of the present study are to evaluate the psychosocial adjustment, including emotional and social skills, of vertically infected HIV pediatric patients receiving HAART in Greece and to assess whether adverse selective and/or global aspects of emotional and social functions are differentially associated with HIV-related complications, such as HIV-related neuroimaging abnormalities.

## Methods

### Study design

A matched case-control study design was applied in order to assess the study objectives. The study protocol was approved by the Human Research and Ethics Committee of the 'Aghia Sofia' Children's Hospital. Informed consent for study participation was requested from all participants' legal guardians prior to study participation.

### Study population

The source population of the case groups consisted of all vertically infected children and adolescents with HIV (n = 24) registered and monitored at the Hellenic National Referral Centre for Pediatric HIV Infection. No exclusion criteria for study participation regarding age, gender, ethnicity, or socioeconomic status were applied. Parental consent for study participation was not provided for one patient (4.1%). One patient (4.1%) died during the course of the study and was thus excluded from all further analyses. Two patients (8.3%) were excluded since they were aged less than 6 months and, consequently, an unreliable assessment of cognitive and social skills was inherent. Hence, the response rate among the remaining study population (n = 20) was 100%.

#### Case groups

The case group consisted of 12 girls (60.0%) and 8 boys (40.0%) aged between 3 through 18 years (mean age 11.76 ± 4.94 years). All study participants received HAART. Upon study initiation, all members of the case group underwent immunological and virological assessments which indicated that the CD4 counts among participants ranged between 394-1858 and 45% (n = 9) of participants had an undetectable viral load. Members of the case group were also subjected to a complete neurological examination and CNS imaging with CT and/or MRI imaging. CNS imaging indicated that 25% (n = 5) of HIV infected children had neuroimaging abnormalities associated with HIV infection. HIV-related neuroimaging abnormalities were defined as brain imaging findings relating to either symmetrical multiple hyperintense signals localized bilaterally in the subcortical white matter or micronodular multifocal calcifications distributed mainly in basal ganglia and cerebellum [7]. In addition, three out of five patients had movement disorders, mainly spasticity of lower extremities. Hence, the following case groups were established: (1) Case Group 1: HIV infected children without neuroimaging abnormalities (n = 15); and, (2) Case Group 2: HIV infected children with neuroimaging abnormalities (n = 5).

#### Control group

The control group consisted of children and adolescents selected from public primary and secondary schools located in Athens, Greece. Eligible participants with a history of speech, language, or communication impairments, as well as other developmental disorders, were excluded from the study. Two controls (n = 40) were selected for each case, and were matched with respect to gender, age (±1 year), and demographic variables, including parental education (i.e. years of education) and profession (i.e. manual, employee, and academic professions).

### Measurements

#### Assessment of Cognitive Development

All study participants underwent a detailed developmental assessment by experienced developmental pediatricians at the Development Assessment Unit of the "Aghia Sophia" Children's Hospital in Athens, Greece. Demographic characteristics and perinatal risk factors known to influence cognitive development were assessed retrospectively. Each child was assessed individually in a secluded room, seated side by side with the developmental paediatrician at a table suitable for the child's height. An identical order of assessments was implemented for all study participants in order to diminish the occurrence of an information bias consequent to fatigue. The cognitive development of study participants aged less than 7 years was assessed with the Griffiths Test, while those aged greater than 7 years was assessed with the Greek standardized version of Wechsler Intelligence Scale III (WISC III) [18-22]. Both tests were utilized accordingly in order to derive General, Practical, and Verbal Intelligence Quotient (IQ) levels, respectively, for study participants. The following cut-off values of IQ scores were applied for all cognitive functions evaluated: (a) normal: 85 < IQ≤120; (b) mild: 70 < IQ≤85; (c) moderate: 50 < IQ≤70; and (d) severe: IQ≤50.

#### Assessment of Psychosocial Adjustment

The age specific Strengths and Difficulties Questionnaire (SDQ) was applied in order to evaluate study participants' psychosocial adjustment, including emotional and social skills. The SDQ is comprised of five components including(1) Emotional Symptoms Score (Normal: 0-5; Borderline: 6; Abnormal: 7-10); (2) Conduct Problems Score (Normal: 0-3; Borderline: 4; Abnormal: 5-10); (3) Hyperactivity Scale (Normal: 0-5; Borderline: 6; Abnormal: 7-10); (4) Peer Problems Scale (Normal: 0-3; Borderline: 4-5; Abnormal: 6-10); and (5) Prosocial Scale (Normal: 6-10; Borderline: 5; Abnormal: 0-4). With the exception of the Prosocial Scale, the sum of the scores derived from the SDQ components was utilized in order to generate the Total Difficulties Score (Normal: 0-15; Borderline: 16-19; Abnormal: 20-40) of study participants [23-25]. The age specific SDQ questionnaire was administered and completed by participants' legal guardians.

### Statistical analysis

The predefined control group was applied as the basis of comparison for all statistical analyses undertaken. The student's t-test was used to compare mean values of continuous variables. Fisher's exact test was used to compare the occurrence of categorical variables, including demographic characteristics and perinatal risk factors, between groups. Ordinal scores of both the IQ and SDQ scales were compared with the Wilcoxon rank sum exact test, respectively. A p-value (*p*) of <0.05 was considered as the significance criterion. Statistical analyses were conducted with SAS version 9.0 (SAS Institute Inc., USA).

## Results

The study population (N = 60) consisted of twenty vertically infected HIV positive children and forty controls matched for age, gender, parental education, and parental profession. The mean age (± SD) of HIV patients was 11.76 ± 4.94 years and did not significantly differ from that of the control group (11.84 ± 4.80 years, p = 0.956). Among the case group (n = 20), 25% (n = 5) had CNS imaging indicative of neuroimaging abnormalities (Table 1). HIV patients without neuroimaging abnormalities did not significantly differ from those with neuroimaging abnormalities with respect to age, treatment regime, years of HAART treatment, and/or laboratory findings, including viral load and CD4 cell counts (Table 2).

**Table 1:** Demographic characteristics of the study population according to the presence of HIV-related neuroimaging abnormalities (NA).

	HIV patients without NA (n = 15)	p-value	HIV patients with NA (n = 5)	p-value	Control group (n = 40)
Age*	12.34 ± 4.58	0.721**^†^**	10.00 ± 6.09	0.437**^†^**	11.84 ± 4.80
Male gender	6 (40.0%)	0.242**^‡^**	2 (40.0%)	0.366**^‡^**	16 (40.0%)
Ethnicity					
Greek	10 (66.7%)	1.00	4 (80.0%)	1.00	40 (100.0%)
Other	5 (33.3%)	< 0.0001**^‡^**	1 (20.0%)	0.111**^‡^**	0 (0.0%)
Attendance in private school	0 (0.0%)	0.099**^‡^**	0 (0.0%)	0.485**^‡^**	9 (22.5%)
Mother's Education*	8.40 ± 2.03	0.360 ^**†**^	8.60 ± 4.83	0.711 ^**†**^	9.15 ± 2.88
Father's Education *	7.28 ± 2.03	0.106 ^**†**^	9.60 ± 2.51	0.965 ^**†**^	8.78 ± 2.41
Mother's Profession		0.189^§^		0.526^§^	
Manual	15 (100.0%)		4 (80.0%)		34 (85.0%)
Employee	0 (0.0%)		0 (0.0%)		3 (7.5%)
Academic	0 (0.0%)		1 (20.0%)		3 (7.5%)
Father's Profession		0,518^§^		0.237^§^	
Manual	12 (80.0%)		1 (20.0%)		25 (62.5%)
Employee	3 (20.0%)		4 (80.0%)		14 (35.0%)
Academic	0 (0.0%)		0 (0.0%)		1 (2.5%)

*Data expressed in mean years ± SD

**^† ^**Student's t-test p-value

**^‡ ^**Fisher's exact test p-value

^§ ^Likelihood ratio exact test

**Table 2:** Comparison of patient characteristics according to the presence of HIV-related neuroimaging abnormalities (NA).

	HIV patients without NA (n = 15)	HIV patients with NA (n = 5)	p-value
Age*	12.35 ± 4.5	10 ± 6.0	0.371^†^
HIV Treatment			
HAART	9 (60.0%)	2 (40.0%)	0.298^‡^
HAART + AZT	6 (40.0%)	3 (60.0%)	-
Age of HAART Initiation*	6.59 ± 3.9	4.3 ± 5	0.306^†^
Months on HAART *	5.75 ± 2.3	5.7 ± 2.5	0.964^†^
Stage			0.026^§^
A1	2 (13.3%)	0 (0.0%)	
B1 or B2 or B3	13 (86.7%)	4 (80.0%)	
C1 or C3	0 (0.0%)	1 (20.0%)	
Viral Load			0.528^§^
< 40	5 (33.3%)	1 (20.0%)	
40-1000	8 (53.3%)	4 (80.0%)	
1000-10 000	2 (13.3%)	0 (0.0%)	
CD4 count			0.140^§^
< 200 cells	1 (6.7%)	2 (40.0%)	
200-500 cells	1 (6.7%)	0 (0.0%)	
> 500 cells	13 (86.7%)	3 (60.0%)	

*Data expressed in mean years ± SD

**^† ^**Student's t-test p-value

**^‡ ^**Fisher's exact test p-value

^§ ^Wilcoxon rank-sum test p-value

### HIV infected children without neuroimaging abnormalities

As indicated in Table 1, 60.0% (n = 9) of HIV infected children without neuroimaging abnormalities were girls. HIV infected children without neuroimaging abnormalities were not found to significantly differ from controls with respect to either maternal or paternal educational and/or professional levels (Table 1).

With regard to perinatal risk factors for impaired cognitive development, only a single case (6.7%) of HIV infected children without neuroimaging abnormalities was found to have been born prematurely. Moreover, HIV infected children without neuroimaging abnormalities were significantly more likely to have experienced hypoxia at birth (Table 3).

**Table 3:** Perinatal risk factors and cognitive function according to the presence of HIV-related neuroimaging abnormalities (NA).

	HIV patients without NA (n = 15)	p-value	HIV patients with NA (n = 5)	p-value	Control group (n = 40)
**Perinatal risk factors**					
Premature Birth	1 (6.7%)	0.434**^†^**	1 (20.0%)	0.332**^†^**	3 (7.5%)
Low Birth Weight	3 (20.0%)	0.205**^†^**	1 (20.0%)	0.374**^†^**	4 (10.0%)
Hypoxia at Birth	4 (26.7%)	0.004**^†^**	3 (60.0%)	< 0.0001**^†^**	0 (0.0%)
**Intelligence Quotient**					
General IQ Score*	82.4 ± 18.8	0.432 ^**‡**^	58.8 ± 11.82	0.027 ^**‡**^	78.0 ± 18.2
Normal (85 < IQ≤120)	6 (40.0%)		0 (0.0%)		10 (25.0%)
Mild (70 < IQ≤85)	3 (20.0%)		1 (20.0%)		16 (40.0%)
Moderate (50 < IQ≤70)	6 (40.0%)		2 (40.0%)		13 (32.5%)
Severe (IQ≤50)	0 (0.0%)	0.721^§^	2 (40.0%)	0.012^§^	1 (2.5%)
Practical IQ Score*	85.3 ± 22.6	0.306 ^**‡**^	57.6 ± 15.0	0.042 ^**‡**^	78.5 ± 21.6
Normal (85 < IQ≤120)	5 (33.3%)		0 (0.0%)		11 (27.5%)
Mild (70 < IQ≤85)	3 (20.0%)		2 (40.0%)		12 (30.0%)
Moderate (50 < IQ≤70)	7 (46.7%)		2 (40.0%)		16 (40.0%)
Severe (IQ≤50)	0 (0.0%)	0.975^§^	1 (20.0%)	0.108^§^	1 (2.5%)
Verbal IQ Score*	80.5 ± 14.9	0.660 ^**‡**^	65.6 ± 14.4	0.083 ^**‡**^	78.4 ± 15.3
Normal (85 < IQ≤120)	6 (40.0%)		0 (0.0%)		12 (30.0%)
Mild (70 < IQ≤85)	4 (30.8%)		1 (20.0%)		12 (30.0%)
Moderate (50 < IQ≤70)	5 (38.5%)		3 (60.0%)		14 (35.0%)
Severe (IQ≤50)	0 (0.0%)	0.848^§^	1 (20.0%)	0.066^§^	2 (5.0%)

* Data expressed as mean score ± SD

**^† ^**Fisher's exact test p-value

**^‡ ^**Student's t-test p-value

^§ ^Wilcoxon rank sum test exact p-value

With respect to the General IQ, while 40.0% (n = 6) of patients without neuroimaging abnormalities were observed to have scores indicative of moderate mental retardation, none had severe mental retardation. In addition, 46.7% (n = 7) of this case group were observed to have Practical IQ scores indicative of moderate retardation. Finally, with regard to the Verbal IQ, in excess of one third of HIV infected children without neuroimaging abnormalities were found to have Verbal IQ scores indicative of moderate retardation. Similarly to the General and Practical IQ scores, none of the HIV infected children without neuroimaging abnormalities were found to have IQ scores indicative of severe retardation (Table 3).

With respect to the SDQ component scores, the overwhelming majority of HIV infected children without neuroimaging abnormalities were found to have normal scores for all components of the SDQ (Table 4). However, HIV infected children without neuroimaging abnormalities were significantly more likely to have both Abnormal Emotional Symptoms and Hyperactivity scores. Furthermore, none of the patients had abnormal scores regarding their social behaviour (i.e. Peer Problems and Prosocial scores). Thus, HIV infected children without neuroimaging abnormalities were observed to experience maladjustment solely with respect to their emotional and activity spheres, while their social skills were unaffected (Table 4).

**Table 4:** Comparison of SDQ component scores according to the presence of HIV-related neuroimaging abnormalities (NA).

SDQ Component Score		HIV patients without NA (n = 15)	p-value	HIV patients with NA (n = 5)	p-value	Control group (n = 40)
**Emotional**	Mean ± SD*	3.4 ± 2.3	0.073^†^	2.2 ± 1.1	0.822^†^	2.4 ± 1.7
	Normal	12 (80.0%)		5 (100.0%)		38 (95.0%)
	Borderline	1 (6.7%)		0 (0.0%)		2 (5.0%)
	Abnormal	2 (13.3%)	0.047^‡^	0 (0.0%)	0.788^‡^	0 (0.0%)
**Conduct**	Mean ± SD*	1.7 ± 1.5	0.533^†^	2.6 ± 0.9	0.255^†^	2.0 ± 1.2
	Normal	13 (86.7%)		4 (80.0%)		35 (87.5%)
	Borderline	1 (6.7%)		1 (20.0%)		4 (10.0%)
	Abnormal	1 (6.7%)	0.838^‡^	0 (0.0%)	0.529^‡^	1 (2.5%)
**Hyperactivity**	Mean ± SD*	4.3 ± 2.9	0.071^†^	2.6 ± 1.7	0.503^†^	3.1 ± 1.6
	Normal	10 (66.7%)		5 (100.0%)		38 (95.0%)
	Borderline	2 (13.3%)		0 (0.0%)		1 (2.5%)
	Abnormal	3 (20.0%)	0.009^‡^	0 (0.0%)	0.788^‡^	1 (2.5%)
**Peer Problems**	Mean ± SD*	2.5 ± 1.6	0.600^†^	2.2 ± 2.4	0.941^†^	2.2 ± 1.3
	Normal	14 (93.3%)		4 (80.0%)		38 (95.0%)
	Borderline	1 (6.7%)		0 (0.0%)		2 (5.0%)
	Abnormal	0 (0.0%)	0.587^‡^	1 (20.0%)	0.033^‡^	0 (0.0%)
**Prosocial**	Mean ± SD*	8.5 ± 1.6	0.630^†^	8.6 ± 1.9	0.699^†^	8.3 ± 1.6
	Normal	14 (93.3%)		5 (100.0%)		36 (90.0%)
	Borderline	1 (6.7%)		0 (0.0%)		3 (7.5%)
	Abnormal	0 (0.0%)	0.921^‡^	0 (0.0%)	0.613^‡^	1 (2.5%)
**Total Score**	Mean ± SD*	11.7 ± 6.6	0.120^†^	9.6 ± 2.6	0.769^†^	9.1 ± 3.6
	Normal	11 (73.3%)		5 (100.0%)		39 (97.5%)
	Borderline	1 (6.7%)		0 (0.0%)		1 (2.5%)
	Abnormal	3 (20.0%)	0.007^‡^	0 (0.0%)		0 (0.0%)

* Data expressed as mean score ± SD

^† ^Student's t-test p-value

^‡ ^Wilcoxon rank sum test exact p-value

### HIV infected children with neuroimaging abnormalities

As shown in Table 1, 40.0% (n = 2) of HIV infected children with neuroimaging abnormalities were boys and the majority (n = 4, 80.0%) were greater than 7 years of age. HIV infected children with neuroimaging abnormalities were not found to significantly differ from controls with respect to either maternal or paternal educational and/or professional levels (Table 1).

Eighty percent (n = 4) of HIV infected children with neuroimaging abnormalities were born following the conclusion of a full-term pregnancy and with normal birth weight. However, HIV infected children with neuroimaging abnormalities were significantly more likely than controls to have experienced hypoxia at birth (Table 3).

In contrast to HIV infected children without neuroimaging abnormalities, none of those with neuroimaging abnormalities presented with normal General, Practical, or Verbal IQ scores. Among HIV patients with neuroimaging abnormalities, 40.0% (n = 2) had scores indicative of moderate mental retardation regarding both the General and Practical IQ scores. Moreover, in contrast to those cases without neuroimaging abnormalities, 40.0% (n = 2) of HIV infected children with neuroimaging abnormalities had scores indicative of severe mental retardation. Also, the majority of HIV infected children with neuroimaging abnormalities (n = 3, 60.0%) had Verbal IQ scores indicative of moderate retardation and 20.0% (n = 1) had respective scores indicative of severe mental retardation (Table 3).

None of the HIV infected children with neuroimaging abnormalities were observed to have either Borderline or Abnormal Total SDQ scores (Table 4). Moreover, with regard to the SDQ component scores, all members of the particular case group examined were observed to have Normal Emotional Symptoms, Hyperactivity, and Prosocial scores. These findings indicate that in contrast to those cases without neuroimaging abnormalities, children with HIV who presented with neuroimaging abnormalities do not appear to have any grade of difficulties regarding either their emotional and/or activity spheres. However, HIV-patients with neuroimaging abnormalities were significantly more likely than their healthy peers to have Abnormal Peer Problems.

## Discussion

Overall, the incidence of HIV infection in Greece (37.9 incident cases per million inhabitants) is markedly lower than that reported in other Western European countries (76.1 incident cases per million inhabitants) [15,26]. The prevalence of HIV infection among pregnant women residing in Greek urban areas (13 per 10,000 women) is approximately half (1.32%) of that reported in other European capitals [8,27]. Specifically, among 1517 pregnant women (1250 of Greek origin and 267 of other ethnicities) surveyed during 1999-2000, 2 were HIV positive. Moreover, the cumulative prevalence of mother-to-child HIV transmissions (MTCT) in Greece was 0.5% and is significantly lower than that reported throughout the WHO European Region [6,10,27]. To date, 24 children born with HIV MTCT currently reside in Greece and are registered at the National Referral Centre for Pediatric HIV infection. Of these, 20 children (83.3%) were assessed for the purposes of the present study. According to surveillance data compiled by the Hellenic Centre of Infectious Diseases Control, MTCT is the most frequent (63.76%) transmission route in children aged less than 13 years. Hence, factors associated with low prevalence rates of HIV infection among pregnant women in the specific region, including free access to HIV screening tests during the first trimester of pregnancy and free access to antiretroviral drugs during pregnancy, may have contributed to the low observed levels of pediatric HIV infection in Greece [27,28].

The prevalence of HIV-related neuroimaging abnormalities among vertically infected children with HIV evaluated for the purposes of our study was 25.0%. The observed prevalence of neuroimaging abnormalities is markedly greater than those reported in the scientific literature, ranging between 1.6% and 10% [3,29]. Prior to the introduction of HAART, the prevalence of neuroimaging abnormalities among HIV infected children ranged between 35% and 50% [4]. The introduction of HAART has contributed to the diminishment of such findings [3]. The elevated prevalence of HIV-related neuroimaging abnormalities among the study population may be attributed to the fact that CNS involvement may be the initial presentation of HIV infection among as many as 18% of pediatric HIV patients [7].

With regard to the presence of perinatal risk factors for impaired cognitive development, the study findings indicated that HIV infected children both with and without neuroimaging abnormalities were as likely as controls to have experienced either premature birth and/or low birth-weight. In contrast, both case groups were more likely to have experienced hypoxia at birth as compared to controls. Although the potential detrimental effects of hypoxia at birth upon consequent childhood development among otherwise healthy neonates have been extensively documented, there exists limited evidence regarding similar effects among neonates with HIV infection [30].

The study findings indicated that only 40.0% of patients without neuroimaging abnormalities had a general IQ score within normal range. Moreover, none of the patients without neuroimaging abnormalities had a General IQ score indicative of severe mental retardation. No statistically significant difference was observed between the Verbal and the Practical IQ scores of HIV patients without neuroimaging abnormalities as compared to their healthy peers. Hence, vertically infected children without neuroimaging abnormalities receiving HAART were observed to have a global intelligence analogous to non-infected children. The study findings are in agreement with those established in the literature [30-32]. Elevated rates of moderate and severe cognitive impairment among children with HIV have been reported [8,10] but are attributed to the lack or limited administration of HAART among the children examined [4,32].

Due to the structural, and consequent functional, alterations inherent in the central nervous system, the effects of HIV-related CNS involvement upon children's cognitive development have been documented [4,6,7]. In the present study, among children with HIV and concomitant neuroimaging abnormalities, none had a General, Practical, and/or Verbal IQ score within the normal range. Moreover, two fifths of the patients in this group had a General IQ score indicative of severe mental retardation. Thus, despite the administration of HAART, children with HIV who presented with neuroimaging abnormalities were observed to have severe deficits in cognitive function as compared to non-infected children.

With respect to the emotional and social skills of HIV infected children, neither those children with nor without neuroimaging abnormalities had SDQ scores indicative of abnormal overall psychosocial maladjustment. This is in agreement with similar findings in the literature [33,34]. Moreover, patients without neuroimaging abnormalities were significantly more likely to have abnormal Emotional and Hyperactivity scores as compared to their healthy peers. Even so, among patients without neuroimaging abnormalities, neither their emotional nor their social skills were affected. These findings, though, may be partly attributed to the fact that patients had not been disclosed of their specific health condition prior to the time of assessment.

In contrast, children with HIV who presented with neuroimaging abnormalities had an elevated frequency of Abnormal Peer Problems, as compared to their healthy counterparts. However, while the social skills of patients with neuroimaging abnormalities were compromised, their emotional adjustment was similar to that of their healthy peers. The impaired verbal skills of children with HIV who presented with neuroimaging abnormalities may lead to compromised communication skills and consequent impaired peer relations [34]. As a result, such impaired functions may be attributed multilaterally to both impaired cognitive and verbal skills.

This study provides evidence suggesting that vertically infected children with HIV receiving HAART are observed to have an increased risk for poor cognitive outcome solely if they present with HIV-related neuroimaging abnormalities. As compared to their healthy peers, pediatric patients without neuroimaging abnormalities are more likely to experience maladjustment with respect to their emotional and activity spheres, while children with HIV who present with neuroimaging abnormalities are more likely to have compromised social skills. The observed normal scores regarding emotional adjustment and social parameters could be attributed either to the fact that systematic psychological and social support is provided by the national health services, or to the fact that all cognitive, emotional, and social skills assessments took place prior to the disclosure of HIV infection among the population examined.

Children who are unable to master adaptive strategies for emotional self-regulation at early ages demonstrate numerous problematic outcomes, including impaired social competence and delayed externalization of problems during late adulthood [35-37]. The present study findings indicating that pediatric HIV patients have an increased likelihood of abnormal emotional and hyperactivity scores may be indicative of a potentially elevated risk for impaired social skills in adulthood, particularly following the additional emotional distress inherently imposed following the disclosure of patients' disease status. As a result, it is recommended that the assessment of both the emotional and social skills of pediatric HIV patients should be undertaken prior to disclosure in order to efficaciously address the further development of either emotional and/or social maladjustment [38].

The strengths of the study include its contribution to the related scientific literature regarding the concomitant assessment of cognitive, emotional, and social function among children with HIV according to the occurrence of HIV-related neuroimaging abnormalities, following HAART. Moreover, while the study population reflects in excess of 83% of all pediatric HIV patients reported in Greece, its limited size may inhibit the generalization of the study findings to larger cohorts, particularly of older age. Due to the cross-sectional study design, the limitations of the study include the inability to establish an etiological relationship between HIV-related neuroimaging abnormalities and impaired cognitive and psychosocial development among pediatric HIV patients receiving HAART. A longitudinal study is necessary in order to assess whether the emotional and psychosocial characteristics of pediatric HIV patients in adulthood may vary, independently of HIV-related neuroimaging abnormalities, following the disclosure of their condition, initiation of sexual relationships, and consequent lifestyle changes.

## Conclusions

HIV infected children without neuroimaging abnormalities are more likely to experience maladjustment with respect to their emotional and activity spheres, while HIV infected children with neuroimaging abnormalities are more likely to present with compromised social skills. Optimal emotional adjustment and social parameters may be attained by systematic psychological and social support provided by the national health services.

## Competing interests

The authors declare that they have no competing interests.

## Authors' contributions

LT contributed to the conception, design, and acquisition of data for the study, and participated in the study coordination. GB contributed to the acquisition of data for the study and helped draft the manuscript. EC performed the statistical analyses, interpretation of the data, and helped draft the manuscript. VS critically revised the manuscript for intellectual content. DK participated in the study coordination and critically revised the manuscript for intellectual content. MT contributed to the conception and design of the study. All authors read and approved the final manuscript.

## Pre-publication history

The pre-publication history for this paper can be accessed here:

http://www.biomedcentral.com/1471-2431/10/99/prepub
